# Editorial: sORF Encoded Peptides in Health and Disease

**DOI:** 10.3389/fgene.2022.878014

**Published:** 2022-03-24

**Authors:** Joseph Rothnagel, Harsha Gowda, Gerben Menschaert

**Affiliations:** ^1^ The University of Queensland, St lucia, QLD, Australia; ^2^ Cancer Research, QIMR Berghofer Medical Research Institute, Herston, QLD, Australia; ^3^ School of Biomedical Sciences, The University of Queensland, Herston, QLD, Australia; ^4^ Faculty of Health, Research–Biomedical Sciences, Queensland University of Technology, Herston, QLD, Australia; ^5^ Ghent University, Ghent, Belgium

**Keywords:** short open reading frames, peptide-regulators, sORF informatics, proteogenomics, sORF-encoded peptides, micropeptides

This research topic covers the recent discovery that biologically relevant peptides of between 6 and 100 or more residues can be directly synthesized by translating ribosomes rather than proteolytic processing of larger precursor proteins. Functional sORF-encoded peptides (SEPs/sPEPs/micropeptides) have been identified across organisms including bacteria, fungi, insects, plants, and mammals. However, their discovery has been and still is impeded by the challenge of isolating these peptides from cellular lysates and distinguishing true SEPs from the proteolytic fragments of larger proteins. SEPs were first identified bioinformatically through analysis of sequence conservation of short open reading frames between species or stochastically by researchers looking at a particular tissue or organism. The field needed a more systematic approach coupled with high throughput methods in order to speed up the identification of these elusive peptides. This wish was granted with the advent of new developments in mass-spectrometry coupled with ribosome profiling; the so called proteogenomics approach. To identify these SEPs using a mass spectrometry-based approach necessarily requires the availability of protein databases containing these short sequences. Ribosome footprints from ribosome profiling could serve as a guide to predict sequence of novel SEPs that can be used to search mass spectrometry data. Kute et al. provide us with an overview of different computational and experimental techniques that can be used for the identification and characterization of small ORFs and their encoded SEPs. Moreover, several assays are described to further validate their physiological functions.

Fijalkowski et al. compared different protein extraction methodologies and subsequent detection of SEPs in the prokaryotic model pathogen *Salmonella typhimurium*. They developed an optimized protocol for the enrichment and detection of SEPs using amphipathic polymer amphipol A8-35 that takes advantage of differential peptide vs. protein solubility. The paper reports identification of 111 of the total 498 annotated SEPs in *S.typhimurium*. They demonstrate the advantage this approach offers compared to conventional proteomics workflows. Methods to overcome poor detection of SEPs due to short length and low abundance has been an impediment for comprehensive mapping of these molecules in all the organisms. This study describes an effective strategy to improve our ability to detect and monitor SEPs. Although the study was done on *S. typhimurium*, the approach should be generally applicable to any sample.

Parmar et al. report on the identification of non-canonical alternative ORFs and translation products thereof using tandem mass spectrometry for the model organism *C. elegans*. A workflow using different enrichment strategies (C8 reverse phase, acid precipitation and Tris-tricine SDS Page and in-gel digestion) and alternative cleavage and its positive impact is reported. Importantly, construction of a more comprehensive search database (allORF) combining both known annotation and predictions based on ribosome profiling (sORFs.org and OpenProt) also increases the detection of SEPs. Finally, the usage of a timsTOF Pro instrument and alternative search engines further improve the detection of novel proteins.

Dib et al. looked at the regulation of a SEP encoding transcript using the *Drosophila* polished rice SEP as an example. Polished rice (*pri*, a.k.a. *tarsal less* or *mille pattes*) encodes four almost identical SEPs (11–32 amino acids in length) that are essential for insect development. The authors build on earlier studies showing regulation of *pri* expression by the insect steroid hormone ecdysone. They went on to identify a complex array of enhancers that direct expression of *pri*, both temporally and spatially, in different tissue types during development.

Proteins are important biomolecules that modulate almost all functions in a cell. Therefore, cataloguing all the proteins encoded in an organism is an important step towards understanding mechanisms that govern its function. Historically, larger proteins that are highly conserved across species were easy to predict. These predicted proteins were annotated in protein reference databases of each organisms that served as a starting point for most biologists to characterize their function. Any proteins that are not annotated in these reference databases are unlikely to be studied by most biologists. There is now sufficient evidence from different organisms that SEPs have been missed by most annotation pipelines. Some researchers have referred to this as dark proteome as they remain unknown. Methodological advances and functional work presented in articles published in this special issue will contribute towards our ability to systematically map these SEPs and characterize their function across different organisms. Importantly, this understanding may in turn lead to novel therapeutic interventions for the treatment of human and non-human diseases by either mimicking endogenous SEPs, or by modulating their activity or of their targets.

